# Thyroid dysfunction among patients assessed by thyroid function tests at a tertiary care hospital: a retrospective study

**DOI:** 10.11604/pamj.2024.49.7.44173

**Published:** 2024-09-04

**Authors:** Emmanuel Donkoh Aidoo, Grace Korkor Ababio, Benjamin Arko-Boham, Emmanuel Ayitey Tagoe, Nii Ayite Aryee

**Affiliations:** 1 Department of Medical Biochemistry, University of Ghana Medical School (UGMS), University of Ghana, Legon, Ghana,; 2 Department of Anatomy, University of Ghana Medical School (UGMS), University of Ghana, Legon, Ghana,; 3 Department of Medical Laboratory Sciences, School of Biomedical and Allied Health Sciences (SBAHS), University of Ghana, Legon, Ghana

**Keywords:** Thyroid dysfunction, spectrum, hyperthyroid, primary hyperthyroidism

## Abstract

**Introduction:**

previous studies in African populations have not extensively described the spectrum of thyroid dysfunction using the profile of thyroid hormones. Although iodine deficiency is a common thyroid disorder in Africa, it does not represent the entire spectrum of thyroid dysfunction seen in patients. This retrospective study aimed to describe the spectrum of thyroid dysfunction among patients seen at the Korle-Bu Teaching Hospital (KBTH), a tertiary care hospital in Accra, Ghana.

**Methods:**

a retrospective analysis of medical records of all consultations on thyroid disorders seen at the Internal Medicine Department of KBTH between January 2019 and December 2021 was conducted. Information on patient demographics, and thyroid hormone profiles (triiodothyronine - FT3, thyroxine - FT4, and thyroid stimulating hormone - TSH) were extracted and subjected to descriptive statistics. The thyroid hormone profiles of the subjects were analyzed and classified into thyroid dysfunction categories using guidelines from the American Thyroid Association (ATA).

**Results:**

out of the 215 patients with thyroid disorders enrolled, 85.1% (n=183) were females and 14.9% (n=32), were males. The mean age of patients was 45±14 years, with most of the patients within the age range of 31-50 years (49.3%; n=106). The most reported thyroid function dysfunction was primary hyperthyroidism (57.7%), followed by primary hypothyroidism (22.3%), subclinical hyperthyroidism (9.3%), euthyroid sick syndrome (6.5%), and subclinical hypothyroidism (4.6%) respectively.

**Conclusion:**

primary hyperthyroidism was the most commonly diagnosed thyroid dysfunction. Hyperthyroidism has been associated with cardiac morbidity and mortality. Timely interventions are required to reduce the morbidity risks and burden associated with the hyperthyroid state.

## Introduction

Globally, thyroid dysfunction has ranked as the second most common endocrine disorder seen in clinical practice, after diabetes [1]. The two main classes of thyroid disorders, hypothyroidism and hyperthyroidism, can be further categorized into clinical and subclinical diseases, based on thyroid hormone levels and clinical presentation [2]. Thyroid disorders affect 200 million people worldwide, according to global estimates [3], with up to 50% of those with thyroid dysfunction, remaining undiagnosed [4]. Thyroid-stimulating hormone (TSH) is critical in the diagnosis of thyroid dysfunction and is affected by age, gender, and iodine nutrition [5]. A wide-ranging spectrum of thyroid dysfunction has been identified by the profile of thyroid function test results. Within the spectrum of thyroid disorders, overt hypothyroidism or hyperthyroidism is usually clinically noticeable, and can be distinguished by the changes in TSH, FT4, and FT3 [2]. However, other thyroid disorders on the spectrum may not be clinically obvious in manifesting their presenting symptoms and clinical signs. These thyroid disorders have been assigned the category, of subclinical thyroid dysfunction (TD) [6]. Subclinical TD comprises both subclinical hypothyroidism and subclinical hyperthyroidism. In both conditions, the thyroid hormone profile presents with serum level of both FT4 and FT3 levels within normal reference limits, while serum thyroid stimulating hormone (TSH) concentration could be mildly elevated or reduced respectively [7,8].

The scope of thyroid diseases that have been repeatedly seen within the African sub-region include hypothyroidism, thyrotoxicosis (which could arise from hyperthyroidism or non-thyroid causes), thyroid malignancies, and iodine deficiency [9]. In many developing countries, the focus in managing thyroid dysfunction has been on treating clinical manifestations. On the contrary, in advanced counties, treatment targets for most forms of thyroid dysfunction, in the absence of pathological disease of the thyroid and pituitary, have mostly evolved from detecting subtle changes in thyroid hormone profiles [10]. In Ghana, limited data exists on the diversity and prevalence of thyroid dysfunction in the general population. Although thyroid disorders are considered a leading cause of endocrine disorders in Africa, the spectrum of non-malignant thyroid disease are yet to be extensively studied in Ghana. This study aimed to determine the spectrum of thyroid dysfunction seen in patients attending a tertiary care hospital in Accra, Ghana over 36 months.

## Methods

**Study design and setting:** this was a hospital-based retrospective study. The study site was the outpatient clinic of the Department of Internal Medicine, Korle Bu Teaching Hospital (KBTH), Accra, Ghana. Ghana is located on the coast of the Gulf of Guinea in West Africa, bordered to the north by Burkina Faso, to the south by the Atlantic Ocean, and the east and west by Togo and Côte d'Ivoire respectively. Ghana´s current population is 32,893,251 [11]. Accra, the capital city where KBTH is located, has a population of 2,660,000 (2023), a 2.11% increase from 2022 [11]. Korle Bu Teaching Hospital remains the leading national referral hospital, and the only tertiary health facility in the southern-eastern part of Ghana. Korle Bu Teaching Hospital also provides support as a Teaching Hospital for students of the University of Ghana Medical School (UGMS) doing their clinical rotation.

**Participants:** electronic medical records of patients with confirmed diagnoses of thyroid disorders seen at the Internal Medicine Department of KBTH between January 2019 and December 2021 were retrospectively analyzed to determine the spectrum of thyroid dysfunction.

**Inclusion criteria:** all medical records of diagnosed cases of thyroid disorders, including patients who presented to the Internal Medicine Department of KBTH hospital with thyroid swellings, and histological confirmation of thyroid disorders were extracted for analysis included in this study.

**Exclusion criteria:** medical records of patients whose case histories were either incomplete, indicated prior use of thyroid medications, or a history of cancer, or had undergone thyroid replacement hormone therapy, or a history of cancer were excluded from analysis.

**Data collection and management:** variables extracted and analyzed were socio-demographic characteristics, and thyroid hormone profiles (serum FT4, FT3, and TSH). Using information extracted from case files, an Excel spreadsheet was created. Based on extracted thyroid hormone values (FT3, FT4, and TSH), patients were categorized into the following groups: subclinical hypothyroidism, hypothyroidism, euthyroid Sick syndrome, subclinical hyperthyroidism, and hyperthyroidism, according to the American Thyroid Association (ATA) guidelines [12].

**Variables:** a total of 6 variables were selected and analyzed (age, gender, FT3, FT4, TSH, and thyroid dysfunction status). Due to incomplete data for some patients´ clinical variables such as presenting symptoms, BMI, and blood pressure, were excluded.

**Bias:** case study files of patients attending the Internal Medicine Department of KBTH between January 2019 and December 2021 were selected randomly. Bias from missing data was minimized by using only complete data of patients. Observer bias was minimized by using student research interns who had no interest in the outcome of the study, to ensure data reliability.

**Sample size:** it was determined using a single population proportion formula with a 95% confidence interval, and a 5% margin of error [13]. Anticipating a hypothyroid prevalence of 16.5% (i.e., p = 0.165) in the population, and grouping all other thyroid dysfunction within the spectrum to be around 84.5%, we arrived at a sample size of 215.

**Statistical methods:** the data was presented in terms of range, mean ± standard deviation (± SD), median, frequencies (on the number of cases), and relative frequencies (percentages) where appropriate. A comparison of quantitative variables between the study groups was done using the student t-test. For the test for associations, the Chi-square (χ^2^) test was performed. Statistical analysis was done using GraphPad Prism version 5.0 (GraphPad Software, and SPSS 22.0 software (SPSS, Inc., Chicago, IL, USA). P<0.05 was considered statistically significant.

**Ethical consideration:** institutional ethical clearance was obtained and approved by the Ethical and Protocol Review Committee of the Community Health Department Review Committee (CHDRC) of the University of Ghana Medical School (UGMS), with approval number, UGMS/CHDRC/034/2022.

## Results

**Participants:** data from medical case files of 215 patients, with confirmed diagnoses of thyroid disorders, between January 2019 and December 2021, were extracted from patient folders and analyzed. Patients were selected irrespective of age and sex. Patient data analyzed were sociodemographic characteristics, and thyroid hormone profiles (serum FT4, FT3, and TSH).

**Descriptive statistics:** out of 215 patients with thyroid disorders enrolled in this study, 85.1% (183) were females, and 14.9% (32) were males. The mean age of the patients was 45±14 years. Most of the patients were within the age range of 31-50 years (49.3%; n=106). Table 1 summarizes the diversity of thyroid disorders diagnosed in this cohort, reporting to the Internal Medicine Department of KBTH during the period under study.

**Table 1:** spectrum of thyroid disorders in the study population

Thyroid status	Frequency, n (%) (N =215)
Primary hypothyroidism	48 (22.3)
Subclinical hypothyroidism	9 (4.2)
Primary hyperthyroidism	124 (57.7)
Subclinical hyperthyroidism	20 (9.3)
Euthyroid sick syndrome	14 (6.5)

N= Total study population; n = frequency of thyroid status

Distribution of patients by thyroid function disorders showed that majority of patients were hyperthyroid (57.7%, n=124). 22.3% (n=48) were diagnosed with hypothyroidism. 9.3% (n=20) and 4.2% (n=9) of patients were diagnosed with subclinical hyperthyroidism and subclinical hypothyroidism respectively. (6.5%, n=14) had euthyroid sick syndrome. The prevalence of thyroid disorders was significantly higher in females than in males (p < 0.05) patients. Analysis of thyroid disorders stratified by age group is presented in Table 2.

**Table 2:** spectrum of thyroid disorders stratified by age group

	Primary hypo (n = 48)	Subclinical hypo (n = 9)	Primary hyper (n =124)	Subclinical hyper (n = 20)	ESS (n = 14)	
Age (mean ± SD) (yrs)	48.1 ± 13.7	58.6 ± 16.6	43.7 ± 13.7	41.3 ± 13.6	48.3 ± 15.2	0.008*
**Age category (yrs): n (%)**						
≤30	6 (12.5)	0 (0.0)	25 (20.2)	4 (20.0)	2 (14.3)	
31 - 50	20 (41.7)	2 (22.2)	63 (50.8)	14 (70.0)	7 (50.0)	0.006*
51 - 70	21 (43.7)	4 (44.5)	31 (25.0)	1 (5.0)	5 (35.7)	
≥71	1 (2.1)	3 (33.3)	5 (4.0)	1 (5.0)	0 (0.0)	

n= subgroup population. Primary hypo = Primary hypothyroidism; Sub hypo = Subclinical hypothyroidism; Primary hyper = Primary hyperthyroidism; Sub hyper = Subclinical hyperthyroidism; ESS = Euthyroid sick syndrome. Fisher exact test was used for proportion comparison. Zero proportions were not included in the calculation.

The mean age for the subclinical hypothyroidism category was 58.6 years, and 41.3 years for subclinical hyperthyroidism. Within the spectrum of thyroid disorders, the mean ages at which each thyroid disorder was diagnosed were significantly different. Within the range of thyroid disorders observed, most patients with hypothyroidism, hyperthyroidism, subclinical hyperthyroidism, euthyroid state, and subclinical hyperthyroidism were within the 31-50 years age bracket, whereas the age for study subjects with subclinical hypothyroidism was 51-70 years. The diversity of thyroid disorders stratified by gender is presented in Table 3.

**Table 3:** spectrum of thyroid disorders in study population stratified by gender

Thyroid disorders	Proportions			Comparison of mean age		
	Female (n = 183)	Male (n = 32)	P-value	Female (n = 183)	Male (n = 32)	P-value
Primary hypothyroidism	40 (21.9)	8 (25.0)	0.739	48.9 ± 13.6	44.5 ± 14.8	0.422
Subclinical hypothyroidism	9 (4.9)	0 (0.0)	-	58.6 ± 16.6	-	-
Primary hyperthyroidism	104 (56.8)	20 (62.5)	0.471	43.4 ± 13.1	44.9 ± 14.1	0.651
Subclinical hyperthyroidism	17 (9.3)	3 (9.4)	1.000	42.5 ± 13.8	34.3 ± 12.7	0.350
Euthyroid sick syndrome	13 (7.1)	1 (3.1)	0.331	47.2 ± 15.2	62.0 ± 0.0	0.368

n= subgroup population; Fisher exact test was used for proportion comparison and Student’s t-test for mean difference with exception of Subclinical hypothyroidism.

Even though the t-test between gender stratification and thyroid spectrum showed no significant difference within gender groupings, there were more females (F-test = 3.54; p- value= 0.008) diagnosed with thyroid function disorders than males (F-test = 11.614; p-value = 0.00001). Most female age subgroups demonstrated a significantly higher prevalence of thyroid function disorders than males of the same age subgroup.

## Discussion

From the results of this study, we found more cases of thyroid dysfunction among females than males. Results from another study in the Central region of Ghana [9], and other countries of Africa, namely Nigeria [14] and Somalia [15] have shown a similar trend. This study also revealed that the mean age at which thyroid dysfunction was diagnosed was 45 years. This conforms with previous studies conducted in the southern part of Ghana, which reported a mean age of 43 years when thyroid dysfunction was first diagnosed [9]. In Nigeria, a mean age of 42 years at diagnosis was reported [14], whereas in Somalia, the reported mean age at diagnosis was 42 years [15]. We found primary hyperthyroidism to be the most prevalent form of thyroid dysfunction (57.7%). Stratified by age, 43.7 ± 13.7 years was the mean age at which, within the spectrum, primary hyperthyroidism was diagnosed. A previous study in the Central region of Ghana revealed that the prevalence of hyperthyroid disorders seen post-1996 was significantly higher than the prevalence pre-1996 (40.0 versus 21.1%, p < 0.001) [9].

Iodine fortification began in Ghana in 1996 when legislation was passed to enforce the iodization of salt [16]. It may appear that the epidemiology and spectrum of thyroid disorders have largely been influenced by the incidence of both excess and deficient levels of iodine, both extremes, posing significant adverse health effects. The percentage of patients from our study, falling into the “hyperthyroid dysfunction” category within the spectrum, is comparable to rates reported in previous studies in Nigeria [14,17], but lower than what was found in a similar study conducted in Somalia [15]. In the first study in Ghana on the clinical presentation of Graves´ disease, a substantial number of patients were hyperthyroid at presentation [18]. It is now known that Graves´ disease is believed to result from a multi-faceted interplay between genetic polymorphisms of various genes, including the TSH receptor [19].

Our study also showed an association between gender and thyroid dysfunction. A systematic review of the etiology of thyrotoxicosis in Africa, found the prevalence of subclinical hyperthyroidism to be less than 1% in areas well supplied by iodine, but as high as 6-10% in iodine-deficient areas [20]. The major limitation of this study was the limited sample size. Larger population studies are needed to determine the true epidemiology and spectrum of thyroid disorders. A retrospective study of thyroid cases during pre- and post-mandatory iodization periods would have been useful to give insights into possible links between the spectrum of thyroid disorders before and after Ghana´s mandatory iodization programme. Nevertheless, our study reiterates the assertion made by Sarfo-Kantanka *et al*. [9] that a progressive increase in the prevalence of hyperthyroid state and autoimmune thyroid disorders may have occurred after the introduction of mandatory iodination in Ghana, and requires attention and post-iodization monitoring.

**Limitations:**this study was limited by the lack of detailed demographic data, co-morbidities, and other clinical variables that could have explained the increasing incidence of thyroid dysfunction. We were unable to retrieve data on thyroid disorders prior to Ghana´s mandatory iodization programme, which began in 1992.

## Conclusion

Primary hyperthyroidism was the most commonly diagnosed form of thyroid dysfunction. This study also showed that more females presented with thyroid diseases, compared to their male counterparts KBTH.

### What is known about this topic

- *A wide spectrum of thyroid dysfunction exists;*
- *The documented prevalence rates of thyroid dysfunction on the African continent are significantly higher in females than males;*
- *Greater awareness about Iodine deficiency disorders and thyroid diseases has resulted in state-sponsored programs that promote salt fortification with iodine*.

### What this study adds

- *This is the first report, outside the boundaries of the central region of Ghana, where a similar study was first conducted, of a high incidence of primary hyperthyroidism being reported as the most common thyroid dysfunction; it further reiterates the observation that thyroid dysfunction may be more common than initially reported;*
- *This study reaffirms the observation made by Sarfo-Kantanka et al. that there could be an increasing trend in hyperthyroid states, particularly after the introduction of mandatory iodination in Ghana;*
- *The results of our study will contribute to generating a registry of thyroid disorders in Africa; although significant improvements have been made in iodine nutrition in Africa, there is no registry of thyroid disorders*.
